# Towards Reverse Vaccinology for Bovine TB: High Throughput Expression of Full Length Recombinant *Mycobacterium bovis* Proteins

**DOI:** 10.3389/fmolb.2022.889667

**Published:** 2022-08-11

**Authors:** Deepa Paliwal, Michelle Thom, Areej Hussein, Divyashree Ravishankar, Alex Wilkes, Bryan Charleston, Ian M. Jones

**Affiliations:** ^1^ School of Biological Sciences, University of Reading, Reading, United Kingdom; ^2^ The Pirbright Institute, Woking, United Kingdom

**Keywords:** bovine tuberculosis, *Mycobacterium bovis*, genome, open reading frame, vaccine, expression, protein purification, PPE

## Abstract

Bovine tuberculosis caused by *Mycobacterium bovis*, is a significant global pathogen causing economic loss in livestock and zoonotic TB in man. Several vaccine approaches are in development including reverse vaccinology which uses an unbiased approach to select open reading frames (ORF) of potential vaccine candidates, produce them as recombinant proteins and assesses their immunogenicity by direct immunization. To provide feasibility data for this approach we have cloned and expressed 123 ORFs from the *M. bovis* genome, using a mixture of *E. coli* and insect cell expression. We used a concatenated open reading frames design to reduce the number of clones required and single chain fusion proteins for protein pairs known to interact, such as the members of the PPE-PE family. Over 60% of clones showed soluble expression in one or the other host and most allowed rapid purification of the tagged bTB protein from the host cell background. The catalogue of recombinant proteins represents a resource that may be suitable for test immunisations in the development of an effective bTB vaccine.

## Introduction

Bovine tuberculosis (bTB) is a major disease throughout the world and is particularly prominent in Africa and parts of Asia. While many developed countries have reduced or eliminated bTB from their cattle populations, major hotspots remain in Europe, America, Canada and New Zealand (Smith, 2012). Phylogenetic analysis of >1,000 non-redundant *M. bovis* genomes has suggested divergence from a last common ancestor with *M. caprae* ∼ 3,500 years ago with spread since aided by animal trade (Zimpel et al., 2020). Control is effected by large scale cattle testing and animals scoring positive are subsequently culled, with an acknowledged impact on sustainability and an increased risk of human infection (Chambers et al., 2014). Various control strategies have been implemented, including those with limited public appeal (Bielby et al., 2014), but it is generally accepted that control in the longer term will require the development of an efficacious cattle vaccine. Multiple potential vaccines have been described (Vordermeier and Hewinson, 2006; Kaufmann et al., 2017; McShane, 2019) but the lead contender for early use remains *Bacillus* Calmette-Guerin (BCG), the cornerstone of human vaccine programmes. However, many of the proteins present in the purified protein derivative (PPD) used in herd screening programs are also present in BCG leading to BCG immunised animals scoring positive, a complication if immunisation and screening occur concurrently. To overcome this, some protein markers, either missing or non-immunogenic in BCG, have been developed to differentiate between infected and vaccinated status (Whelan et al., 2010; Vordermeier et al., 2011; Srinivasan et al., 2019). An alternate approach, to develop a BCG based vaccine that does not lead to positivity in the screening test has also been investigated (Chandran et al., 2019). Although BCG derives from *M*. *bovis*, comparative genomics has revealed genomic losses in the variously passaged BCG stocks when compared to ancestral stocks (Zhang et al., 2013) suggesting that some BCG vaccine stocks may be incapable of providing suitable levels of protection unless supplemented with additional proteins representing the missing ORFs. A number of candidates have been investigated in this respect, including Ag85, ESAT-6, CFP-10 and members of the PPE family (Li et al., 2015; Dai et al., 2017; Tkachuk et al., 2017; Stylianou et al., 2018) although generally as single antigens rather than as a mixture of candidates. As a result, the case has also been made that current candidate vaccines lack diversity and that a broader range of proteins should be considered (Fletcher and Schrager, 2016).

Reverse vaccinology (Rappuoli, 2001; Serruto et al., 2012; Rappuoli et al., 2016) assumes that molecules on the cell surface of a target organism include those that may act as an effective vaccine even if they do not raise a significant serum response following natural infection. The availability of whole bacterial genomes, suitable bioinformatics and high throughput protein expression technologies allows this concept to be tested, with some notable successes (Muruato et al., 2017; Masignani et al., 2019). The feasibility of this approach is in part determined by how many and how easily bTB proteins can be expressed and purified. For *Mycobacterium tuberculosis*, progress in multiple protein expression has been made by the TB Structural Genomics Consortium, although the proteins selected were based primarily on their suitability as drug targets (Chim et al., 2011). To assess feasibility for proteins that might be targeted for vaccine use and to provide such proteins for testing we report a high throughput expression approach (Aricescu et al., 2006; Nettleship et al., 2010; Bird et al., 2014) to the expression of many full-length *Mycobacterium bovis* ORFs.

## Materials and Methods

### Selection of Vaccine Candidates

Candidate bTB proteins were selected by reference to lists derived from published bioinformatics approaches (e.g. (He et al., 2010; Monterrubio-López et al., 2015)) as well as current web sources (Kapopoulou et al., 2011; Dhanda et al., 2016). Throughout the study the genome of *Mycobacterium bovis* strain AF2122/97 was used as the reference strain. Proteins with a range of putative functions related to vaccine use were included, for example roles in virulence, predicted surface expression, previous use as a candidate vaccine and roles in the immune response and immune evasion.

### Molecular Cloning

Sequences representing the candidate ORFs were ordered as custom designed double stranded DNA fragments (Integrated DNA Technologies, Belgium). Sequence predicted to encode transmembrane domains was removed and synthetic genes were codon optimised for *Spodoptera frugiperda* (Sf9) or *E. coli* to maximise expression in the relevant host. All DNA fragments were flanked with 18 base pairs of sequence homologous to the vector pTriEx 1.1 (EMD Biosciences), suitable for expression in *E. coli* under control of the T7 promoter or insect cells under control of the baculovirus very late P10 promoter. All clones were generated by Gibson assembly (Gibson et al., 2009). The sequence designs were such that translation initiated at the bTB ORF initiation codon and fused the C-terminus with the histidine tag present in the vector, except when otherwise indicated. The pTriEx-1.1 cloning vector was linearised by digestion with restriction enzymes *Xho*I and *Nco*I (Thermo Fisher Scientific, Paisley, United Kingdom) and 20 ng gene fragment was assembled into 50 ng linearized pTriEx-1.1 cloning vector using the NEBuilder HiFi DNA Assembly Protocol (NEB, United Kingdom). Clones were isolated following transformation of Stellar competent cells (Takara Bio, France) with confirmation by colony PCR followed by DNA sequencing.

### *E. Coli* Expression

The default strain for protein expression in *E. coli* was SoluBL21 (Kim et al., 2015). Alternate hosts were *E. coli* T7 Express lysY (NEB) and LOBSTR (Andersen et al., 2013) which were used for low yielding constructs. Initial screening was achieved with cultures of ∼20 ml and scaled according to the outcome. Cells were grown at 37°C to an OD_600_ = 0.6 and induced by the addition of IPTG to 0.1 mM. After induction, growth was continued for 4 h at 37°C. Cultures were harvested by centrifugation at 4,000 × *g* for 15 min and disrupted for gel analysis or purification as required. Solubility was determined after lysis in 1% Triton-X detergent and gel analysis of the soluble and pellet fractions. Poor solubility was corrected, where possible, with low temperature induction (16°C) or by varying the IPTG concentration used for induction. Intractable expression was abandoned in favour of alternate constructs or the alternate host.

### Recombinant Baculovirus Expression

Recombinant baculoviruses were produced by co-transfection of Sf9 cells with transfer vectors and linearised baculovirus DNA as described (Porta et al., 2013). Transfections were done in 6 well tissue culture plates at a monolayer confluency of 50%. Viruses were passaged when significant cytopathic effect was observed and viral stocks were amplified to high titre, typically 3 passages, prior to use for infection and protein detection. All viral stocks were produced in Sf9 cells. For protein expression, 100 ml suspension cultures of *AoTni*38 cells (Hashimoto et al., 2012) were infected at a density of 2 × 10^6^ per ml with 10 ml of high titre (>10^7^ pfu per ml) virus stock and the culture continued for 3 days. Cells were harvested by centrifugation and processed as described.

### SDS PAGE

Protein samples were prepared using NuPAGE loading buffer (Fisher Scientific UK Ltd., United Kingdom) and heated to 98°C for 10 min before loading the gel. Proteins were separated by SDS-PAGE using 4–12% precast Tris-Glycine SDS polyacrylamide gels (Fisher Scientific UK Ltd., United Kingdom) for 30 min at 200 V. SDS-PAGE loading used the equivalent of 50 μL of bacterial culture or 5 × 10^4^ insect cells per lane of a 10 lane, 10 cm gel. After electrophoresis, gels were subjected to either Coomassie Brilliant Blue R250 staining or transferred to a polyvinylidene difluoride (PVDF) membrane for western blot analysis.

### Western Blot

Proteins were transferred electrophoretically to PVDF membranes (Immobilon^®^, Merck, Germany) by semi-dry transfer. Membranes were blocked with Protein-Free T20 (TBS) Blocking Buffer (Fisher Scientific UK Ltd., United Kingdom) for 1 h. All subsequent incubations and washes were done in Tris-buffer saline containing 0.1% Tween 80 (TBST). Membranes were incubated with monoclonal Anti-6X His tag^®^ antibody conjugated to HRP (Abcam, United Kingdom) at 1:2000 dilution followed by three washes of 5 min each. The membrane was finally washed with TBS and antibody reactive bands were revealed using ECL Western Blotting Detection Reagent (Geneflow, United Kingdom) and imaged using a Syngene G-Box (Syngene, United Kingdom).

### Purification

*E.coli* cell pellets were lysed in 10% of the original culture volume in a lysis buffer (500 mM NaCl, 20 mM Sodium phosphate buffer pH 7.4, 20 mM imidazole, 1% Triton X-100) supplemented with 1% lysozyme, 1 mM phenylmethanesulfonyl fluoride and 1 mM benzamidine hydrochloride and incubated for 10 min at 4°C. Samples were sonicated for 10 min on ice followed by centrifugation at 15,000 × *g* for 10 min. Infected insect cells were disrupted similarly but without lysozyme in the lysis buffer. Expressed proteins, with an affinity tag of six consecutive histidine residues, were purified by His-mag sepharose Ni beads (GE Life Sciences) using 200 µL bead slurry per preparation. Beads were incubated with the clarified lysate for a minimum of 2 h on a blood wheel before washing and elution according to the vendor’s instructions. For proteins that demonstrated a tendency to insolubility the lysis buffer also contained 34 mM SDS and the wash and elution buffers included 0.1% sodium sarkosyl (Schlager et al., 2012). For secreted proteins the clarified supernatant was adjusted to 0.5 mM nickel sulphate and used directly for IMAC pull-down. Constructs that were positive for expression but failed purification by one of these routes were abandoned.

## Results

### Identification and Generation of Sequences Encoding Putative Vaccine Candidates

**FIGURE 1:**
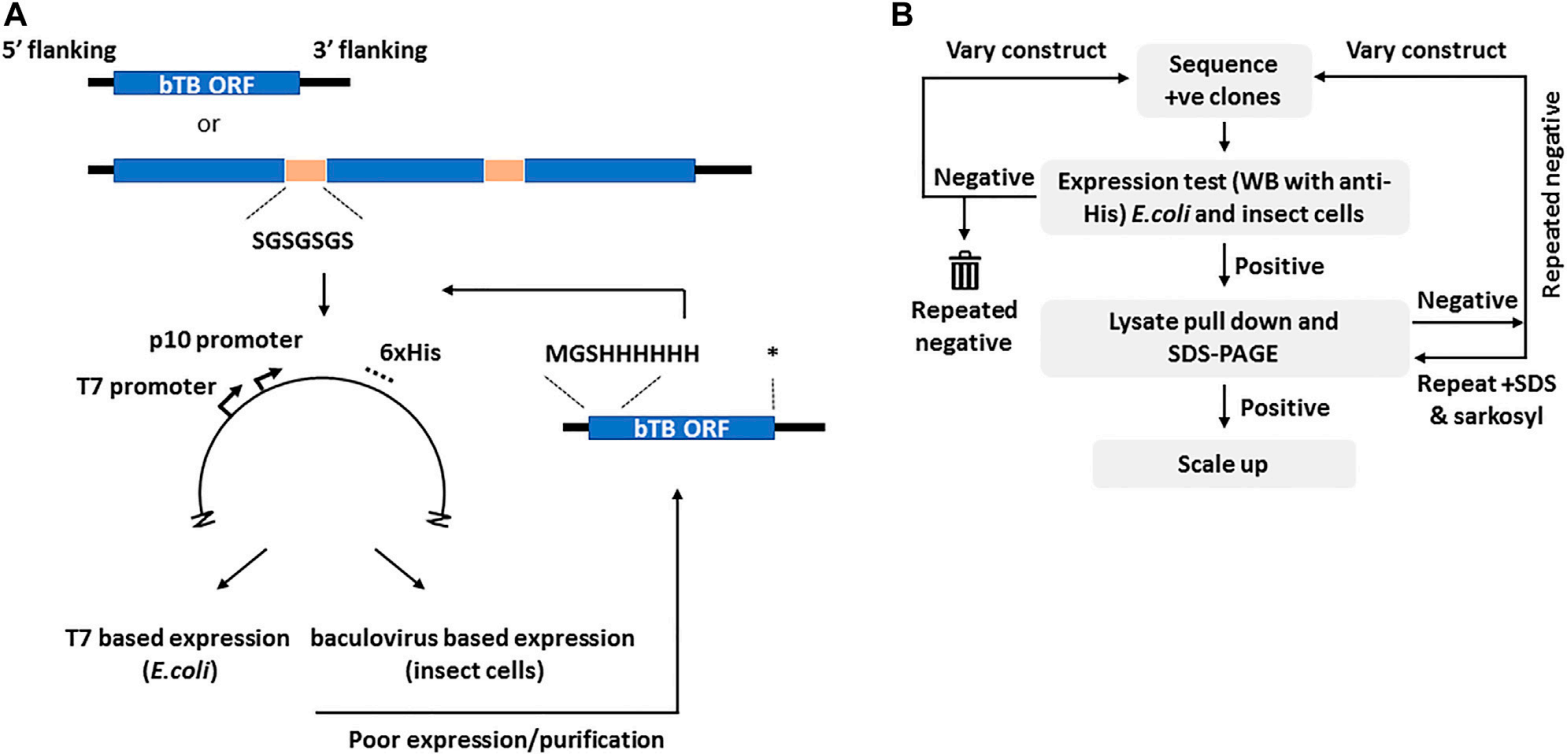
The cloning and selection strategies for the generation of the M. bovis recombinant protein atlas. Panel **(A)**. Generic construct design showing the use of either complete or concatenated ORFs tagged at the C-terminus with polyhistidine as present in vector pTriEx1.1. In the event of poor expression the same ORFs were re-cloned with an N-terminal His tag as shown. The sequence of the flexible linker is indicated. Asterisk–stop codon. Panel **(B)**. The protein expression and purification regimen showing the iterative nature of the process. The varied constructs are those with alternate His tags shown in **(A)**. Initial screening was by Western blot for the His tag in all cases.

The selection of candidates for this study was based on one or more vaccine related properties, viz. their predicted biological function (cell surface protein, virulence or adhesion), predicted immunogenicity, previous use as a candidate vaccine or sequence conservation in multiple mycobacterial genomes. From these considerations the final ORFs selected were grouped into seven loose descriptive categories (Table 1). For ORFs representing complete proteins with a predicted molecular mass of greater than ∼15 kDa, synthetic DNA was produced to the entire coding region with flanking regions that ensured a unique initiator ATG downstream of the Shine-Dalgarno sequence and fusion with a sequence encoding polyhistidine at the carboxyl terminus of the expressed protein, both features of the vector used (Figure 1A). If a signal peptide was present in the original *M. bovis* ORF, assessed by routine submission to the SigP server (Nielsen et al., 2019), it was changed to that of honeybee melittin, known to be efficiently processed in insect cells (Tessier et al., 1991). Sequences predicted to encode transmembrane domains were deleted. In cases where expression of the recombinant protein was either poor or, more usually, insoluble, the design was changed to incorporate an N-terminal polyhistidine sequence after the initiator codon and a stop codon prior to the vector encoded polyhistidine sequence (Figure 1A).

**TABLE 1:** The bTB ORFs selected for recombinant expression tests.

		Expression host	Soluble expression	Purification yield
**Cell wall and cell processes**				
Mb0923	Outer membrane protein Omp A	*E. coli & Insect*	+++	+++
Mb0485	Iron-regulated heparin binding hemagglutinin hbha	*E. coli & Insect*	+++	+++
Mb0419c	Probable glutamine-binding lipoprotein glnh (glnbp)	*E. coli & Insect*	++	+
Mb3840	Exported repetitive protein precursor PirG	*E. coli & Insect*	+++	+
Mb0348	Isoniazid inductible gene protein iniB	*E. coli & Insect*	+++	+++
Mb2293	Probable lipoprotein lppN	*E. coli*	+++	+++
Mb2807c	Probable lipoprotein lppU	Insect	+++	+++
Mb0598c	Probable lipoprotein lpqN	*E. coli & Insect*	+++	+++
Mb1260	Probable lipoprotein lpqX	Insect	+++	+++
Mb1568c	Probable lipoprotein lprI	*E. coli & Insect*	+++	+++
Mb1891c	Alanine and proline rich secreted protein APA	*E. coli & Insect*	+++	+++
Mb3905	6 kda early secretory antigenic target esxa (Esat-6)	*E. coli & Insect*	+++	+++
Mb3904	10 kda culture filtrate antigen esxb (lhp) (cfp10)	*E. coli & Insect*	+++	+++
Mb0296	Low molecular weight antigen 7 esxh (10 kda antigen) (cfp-7)	Insect	+++	+++
Mb0295	Esat-6 like protein esxg	Insect	+++	+++
Mb1229	Esat-6 like protein esxk (Esat-6 like protein 3)	*E. coli & Insect*	+++	+++
Mb1230	Putative Esat-6 like protein esxi (Esat-6 like protein 4)	Insect	+++	+++
Mb1820	Esat-6 like protein esxm	*E. coli & Insect*	+++	+++
Mb1821	Putative Esat-6 like protein esxn (Esat-6 like protein 5)	Insect	+++	++
Mb3475c	Esat-6 like protein esxu	*E. coli & Insect*	+++	+++
Mb3911c	Proteolytic substrate protein espb	*E. coli & Insect*	++	++
Mb0674	Possible ribonucleotide-transport ATP-binding protein	*E. coli & Insect*	+++	++
Mb1036	Probable resuscitation-promoting factor rpfb	*E. coli & Insect*	++	-
Mb1445	Aminoglycosides/tetracycline-transport integral membrane protein	*E. coli & Insect*	++	-
Mb3070	Probable FeIII-dicitrate-binding periplasmic lipoprotein fecb	*E. coli*	++	++
Mb3338	Acid phosphatase sapm	*E. coli & Insect*	++	++
Mb2653c	Probable conserved transmembrane protein	*E. coli & Insect*	-	-
Mb3646c	Esx-1 secretion-associated protein, espa	*E. coli & Insect*	+++	+
Mb3645c	Esx-1 secretion-associated protein espc	*E. coli & Insect*	+++	+++
Mb3644c	ESX-1 secretion-associated protein espd	*E. coli & Insect*	-	-
Mb2002c	Immunogenic protein mpt64 (antigen mpt64/mpb64)	*E. coli & Insect*	+++	+++
Mb1762	Probable conserved transmembrane protein	*E. coli & Insect*	0	++
**Information pathway related proteins**				
Mb0055	Single-strand binding protein Ssb	*E. coli & Insect*	+	+
Mb0671	50s ribosomal protein l7/l12 rplL (sa1)	*E. coli & Insect*	+++	+++
Mb0704	Probable iron-regulated elongation factor tu tuf	*E. coli & Insect*	++	+
Mb0670	50s ribosomal protein l10 rplJ	*E. coli & Insect*	+++	++
Mb1656	30s ribosomal protein s1 rpsA	*E. coli & Insect*	+++	+++
**Virulence related proteins**				
Mb3441	Possible antitoxin VapB47	Insect	+++	+++
Mb2891	Toxin RelG	*E. coli & Insect*	+++	-
**PE/PPE family proteins**				
Mb2548	PE family PE26	Insect	+	+
Rv3622c	PE family protein PE32	*E. coli & Insect*	+++	+++
Mb0293	PE family protein PE5	*E.coli & Insect*	+++	+++
Mb0294	PE family protein PE4	*E.coli & Insect*	+++	+++
Mb3902	PE family-related protein PE35	*E.coli & Insect*	+++	+++
Mb3922c	PE family protein PE36	*E.coli*	+++	+++
Mb3504	PE family protein PE31	Insect	+++	+++
Mb0940c	PE family protein PE7	Insect	+++	+++
Mb1835	PE family protein PE20	Insect	+++	+++
Mb1421	PE family protein PE15	*E.coli & Insect*	+++	+++
Mb3505	PE family protein PPE 60	*E.coli & Insect*	+++	+++
Mb1069c	PE family protein PE 8	*E.coli & Insect*	+++	+++
Mb2457c	PE family protein PE 25	*E.coli & Insect*	+++	+++
Mb1202c	PE family protein	*E.coli & Insect*	+++	+++
Mb3903	PPE family protein PPE68	*E.coli & Insect*	+++	+++
Mb3921c	PPE family-related protein PPE69	*E.coli & Insect*	+++	+++
Mb0939c	PPE family protein PPE 14	*E.coli & Insect*	+++	+++
Mb1836	PPE family protein PPE 31	*E.coli & Insect*	+++	+++
Mb1068c	PPE family protein PPE15	*E.coli & Insect*	+++	+++
Mb1422	PPE family protein PPE 20	Insect	+++	+++
Mb2456c	PPE family protein PE41	*E.coli & Insect*	+++	+++
Mb1228	PPE family protein PPE 18	*E.coli & Insect*	+++	+++
Mb1200c	PPE17 (part)	*E.coli & Insect*	+++	+++
Mb1201c	PPE 17 (part)	*E.coli & Insect*	+++	+++
**Intermediary metabolism & respiration related proteins**				
Mb3871	Bacterioferritin BfrB	*E.coli*	+++	+++
Mb2981	Possible glycosyl transferase	*E.coli & Insect*	++	+
Mb0130	probable serine protease pepA	*E.coli & Insect*	++	++
Mb1272	Probable malate dehydrogenase mdh	*E.coli & Insect*	++	-
Mb0977	Probable succinyl-CoA synthetase (a chain) sucD	*E.coli & Insect*	++	++
Mb3652	Inorganic pyrophosphatase PPA	*E.coli & Insect*	+++	
Mb1129c	Fructose 1,6-bisphosphatase glpX	*Insect*	++	-
Mb3412	Diterpene synthase	*E.coli & Insect*	++	++
**Conserved hypotheticals & unknown proteins**				
Mb2315c	Hypothetical protein	Insect	+	-
Mb3935c	Putative Esat-6 like protein esxf (Esat-6 like protein 13)	*E. coli & Insect*	+++	+++
Mb3920c	Possible Esat-6 like protein esxd	*E. coli & Insect*	+++	+++
Mb3934c	Putative Esat-6 like protein esxe (Esat-6 like protein 12)	*E. coli & Insect*	+++	+++
Mb1066c	Putative Esat-6 like protein esxI (Esat-6 like protein 1)	Insect	+++	+++
Mb1067c	Esat-6 like protein esxj	*E. coli & Insect*	+++	+++
Mb2375c	Putative Esat-6 like protein esx0 (Esat-6 like protein 6)	Insect	+	+
Mb3042c	Esat-6 like protein esxq (tb12.9) (Esat-6 like protein 8)	Insect	+	+
Mb3045c	Secreted Esat-6 like protein esxr (Esat-6 like protein 9)	*E.coli & Insect*	+++	+++
Mb3046c	Esat-6 like protein esxs	*E.coli & Insect*	+++	+++
Mb3474c	Putative Esat-6 like protein esxt	*E.coli & Insect*	+++	+++
Mb3919c	Esat-6 like protein esxc (Esat-6 like protein 11)	Insect	+	-
Mb0959	Periplasmic phosphate-binding lipoprotein PstS1	*E. coli & Insect*	+++	+++
Mb1858	Conserved protein with fha domain, gara	*E. coli & Insect*	++	++
Mb1868c	Malate synthase G GlcB	*E. coli & Insect*	++	++
Mb1943c	Catalase-peroxidase-peroxynitritase T KatG	*E. coli & Insect*	++	++
Mb2006c	Probable cutinase precursor CFP21	*E. coli & Insect*	++	+++
Mb2057c	Stress protein induced by anoxia	*E. coli & Insect*	++	+++
Mb2244	Glutamine synthetase GlnA1	*E. coli & Insect*	++	+++
Mb2898	Cell surface lipoprotein Mpt83 (lipoprotein P23)	*E. coli & Insect*	++	+++
Mb2900	Major secreted immunogenic protein Mpt70	*E. coli & Insect*	++	++
Mb0169	Conserved protein TB18.5	*E. coli & Insect*	+	+
Mb0418c	Serine/threonine-protein kinase PknG	*E. coli & Insect*	+++	+++
Mb2477c	Probable resuscitation-promoting factor RpfE	*E. coli & Insect*	++	-
Mb1319	Conserved protein	*E. coli & Insect*	++	++
Mb1596	Involved in biotin biosynthesis	*E. coli & Insect*	++	++
Mb2656	Universal stress protein family protein TB31.7	*E. coli & Insect*	++	++
Mb3046c	Esat-6 like protein EsxS	*E. coli & Insect*	++	++
Mb2982c	Possible glycosyl transferase	*E. coli & Insect*	++	+
Mb0455c	Cyclopropane fatty acid synthase	*E. coli & Insect*	+++	++
Mb2054c	pfkb	*E. coli & Insect*	+++	+++
Mb3157c	devR	*E. coli & Insect*	+++	+++
Mb2970c	Probable conserved lipoprotein LppX	*E. coli & Insect*	++	
Mb0891	Possible resuscitation-promoting factor rpfA	*E. coli & Insect*	+++	++
Mb1916	Probable resuscitation-promoting factor rpfC	*E. coli & Insect*	++	++
Mb2410	Probable resuscitation-promoting factor rpfD	*E. coli & Insect*	++	++
Mb3274c	Two component sensory transduction transcriptional regulatory protein mtrA	*E. coli & Insect*	+++	+++
Mb0463c	Conserved protein	*E. coli & Insect*	+++	+++
Mb3641	Hypothetical arginine and proline rich protein	*E. coli & Insect*	++	-
Mb0062	Hypothetical protein	*E. coli & Insect*	++	++
Mb1843	Conserved protein	*E. coli & Insect*	++	++
Mb3743c	Conserved protein	*E. coli & Insect*	+++	+++
Mb1833c	Conserved protein	Insect	+++	+++
Mb0854c	Conserved protein	*E. coli & Insect*	+++	+++
Mb2058	Conserved protein Acg	*E. coli & Insect*	++	-
Mb0337c	hypothetical protein	*E. coli & Insect*	++	++
Mb2660c	Conserved protein	*E. coli & Insect*	++	-
Mb 2659	hypoxic response protein 1 hrp1	*E. coli*	+++	+++
Mb3707	Probable bifunctional membrane-associated penicillin-binding protein 1a/1b pona2	*E. coli & Insect*	++	+
Mb0014c	Transmembrane Serine/therorine protein Kinase-B (pknb)	*E. coli & Insect*	++	+++
Mb0979	Probable conserved Transmembrane protein	*E. coli & Insect*	++	+++
Mb0448	GROEL protein-2	*E. coli & Insect*	++	+++

Soluble expression levels: +++ - strongest band on SDS-PAGE, ++ - among the stronger bands, + - visible band, − no visible band. Purification yields: +++ ∼1 mg/L, ++ ∼0.1 mg/L, + <0.1 mg/L, - not purified. Greyed boxes required wash and elution buffers with 0.1% sodium sarkosyl.

More than half of the expressed proteins that were problematic in recombinant form with a C-terminal polyhistidine were improved by this strategy, as described below. For proteins that were <15 kDa, or for proteins whose database entry did not contain an initiator methionine, for example some members of the *esx* family, sequences were concatenated before synthesis so that several ORFs were expressed together, connected by a Gly-Ser linker of 7 residues (Figure 1A). Similarly, for protein pairs such as the proline-proline-glutamate (PPE) and proline-glutamate (PE) complexes, the matching pair was synthesised as a single chain also connected by a flexible linker. The available crystal structures of such complexes show the C-terminus of the PPE partner lying close to the N-terminus of the paired PE (Strong et al., 2006), a distance easily spanned by the added linker. Some *M. bovis* ORFs were persistently difficult to express in either *E. coli* or insect cells and in these cases workaround strategies involved fusion with solubility tags including GFP, SUMO or the B1 domain of streptococcal protein G (GB1) (Lindhout et al., 2003; Pengelley et al., 2006; Lee et al., 2008). Some of these strategies improved expression or purification for some of the ORFs but none provided a universal route to improvement.

### Protein Expression and Purification

**FIGURE 2:**
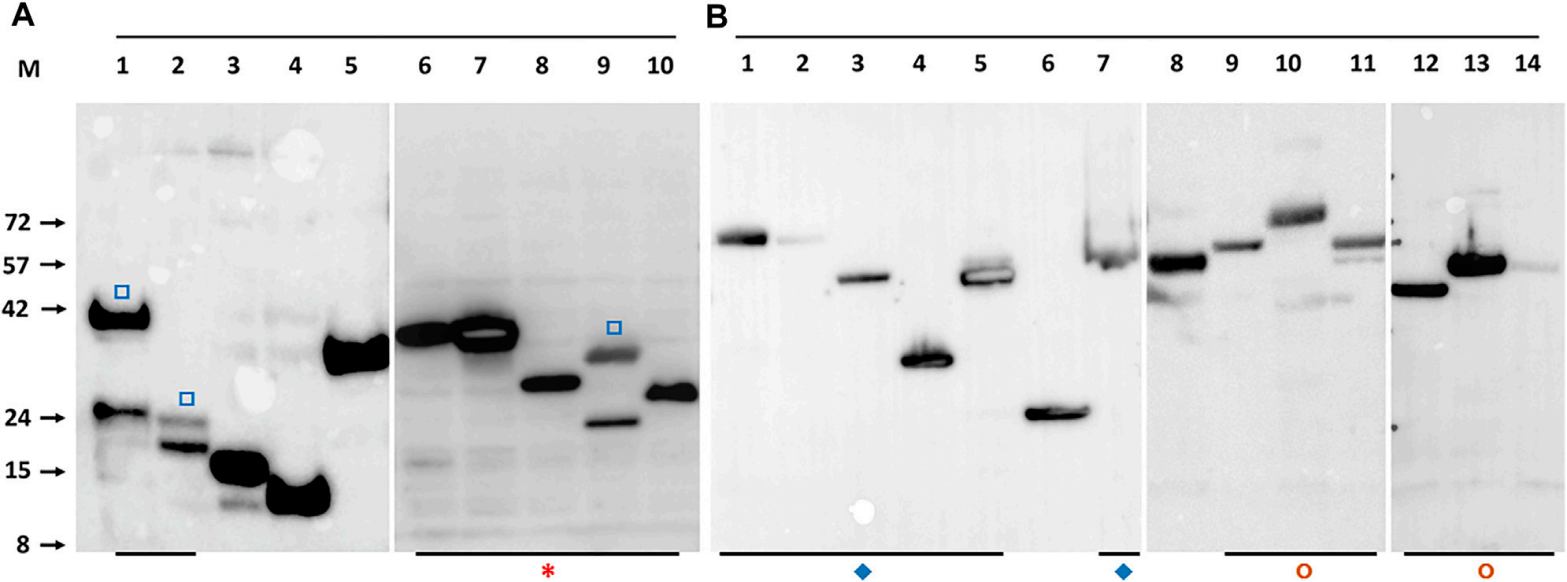
Example Western blot detection of recombinant *M. bovis* proteins with anti-His antibody. Panel **(A)**. Detection of *M. bovis* ORFs expressed in induced *E.coli*. The lane identity and predicted molecular mass are: 1-Mb1228 (39 kDa), 2-Mb1916 (18 kDa), 3-Mb2410 (15 kDa), 4-Mb3441 (11 kDa), 5-Mb3412c (34 kDa), 6-EsxRST (32 kDa), 7-EsxNOQ (30 kDa), 8-EsxU (24 kDa), 9-EsxFDE (27 kDa), 10-EsxAB (21.2 kDa). Panel **(B)**. Detection of *M. bovis* ORFs expressed in baculovirus infected insect cells. The lane identity and predicted molecular mass are: 1-PPE15_PE8 (66 kDa), 2-PPE20_PE15 (67 kDa), 3-PPE31_PE20 (51 kDa), 4-PPE41_PE25 (34 kDa), 5-PPE60_PE31 (53 kDa), 6-MTRA (28 kDa), 7-PPE14_PE7 (53 kDa), 8- Mb0463_Mb0674 (59 kDa), 9-Mb0854_Mb0977 (63 kDa), 10-Mb1036_Mb1129c (74 kDa), 11-Mb1272_Mb2477c (54 kDa), 12-Mb0891c (34 kDa), 13- Mb3070_Mb3641 (54 kDa), 14-Mb3338_Mb3644c (54 kDa). Open square (□) symbols indicate protein which show some breakdown as indicated by at least 2 His antibody reactive bands. Asterisk (*) indicates all Esx related proteins, most as concatenates. Diamonds (♦) indicate concatenated PPE-PE pairs. Circles (◘) other protein concatenates. M indicates the marker track, the molecular masses of which are given on the left of panel **(A)** in kilodaltons.

To maximise the possibility of efficient expression of a diverse range of selected *M. bovis* ORFs as soluble proteins, two different expression systems were used, *E. coli* and recombinant baculoviruses. Screening of the clones or recombinant viruses was done in small scale cultures by Western blot with a directly conjugated antibody recognizing the polyhistidine tag (Figure 1B). The default result was a band of the molecular mass predicted for the translated protein but occasionally the band identified by the Western blot had bands additional to the predicted mass (marked in Figure 2A). We assumed smaller sizes were the result of protein degradation, with the residual C-terminal His tagged fragment being detected, whereas larger proteins were post-translationally modified or oligomeric. Notably, the concatenated PPE-PE complexes gave rise to stable products of the predicted molecular mass (marked in Figure 2B) as did the Esx concatenates (marked in Figure 2A), some of which have been reported elsewhere (e.g., (Li et al., 2016; Mearns et al., 2017)). Concatenated constructs Mb1036_Mb1129c, Mb1272_Mb2477c, Mb3070_Mb3641 and Mb3338_Mb3644c also showed predominantly single bands at the predicted molecular mass when probed with an anti-His antibody (marked in Figure 2B).

**FIGURE 3:**
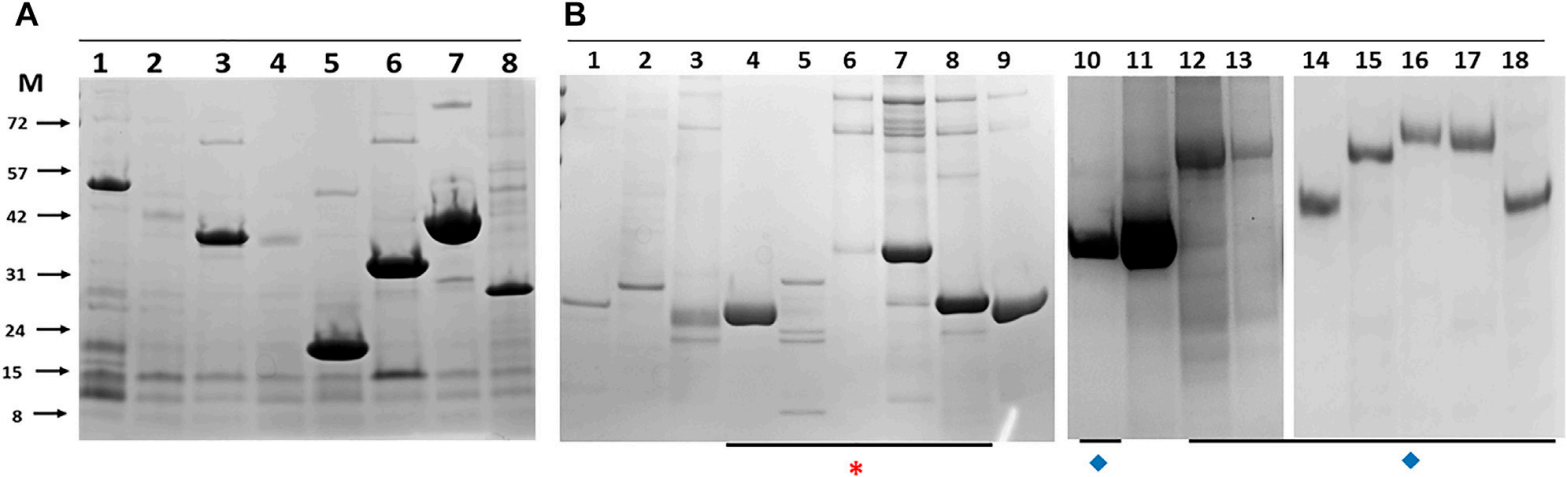
Example histidine-tagged *M. bovis* proteins purified by immobilised metal affinity chromatography pull down with magnetic beads from *E.coli* **(A)** and insect cells **(B)**. The lane identity and predicted molecular masses are: Panel A, 1-Mb0704 (44 kDa), 2-Mb3070 (37 kDa), 3-Mb2054 (36 kDa), 4-Mb2002_Mb0062 (35 kDa), 5-Mb0671 (17 kDa), 6-Mb0854 (31 kDa), 7- Mb3157c_Mb3743c (37 kDa), 8-Mb0337 (26 kDa). Panel B, 1-PE5_PE32 (19.5 kDa), 2-LpqN (25 kDa), 3-LpqX (22 kDa), 4-EsxAB (21.2 kDa), 5-EsxFDE (27.3 kDa), 6-EsxNOQ (30 kDa), 7- EsxRST (32 kDa), 8-EsxU (24 kDa), 9- Mb0485 (21 kDa), 10-PPE41_PE25 (34 kDa), 11-Mb1228 (39 kDa), 12-PPE69_PE36 (46.6 kDa), 13- PPE68_PE35 (47.5 kDa), 14-PPE14_PE7 (51 kDa), 15-PPE4_PE5 (67 kDa), 16- PPE15_PE8 (66 kDa), 17- PPE20_PE15 (67 kDa), 18-PPE31_PE20 (51 kDa). Asterisk (*) indicates all Esx related proteins, most as concatenates. Diamonds (♦) indicate concatenated PPE-PE pairs. M indicates the marker track, the molecular masses of which are given on the left of panel **(A)** in kilodaltons.

Clones positive for expression by western blot were assessed for purification by pull-down from detergent lysates using IMAC magnetic beads and the eluates assessed directly by SDS-PAGE and gel staining (Figure 3). The default outcome was a predominant band of the predicted molecular mass that also agreed with the mass identified by western blot with an anti-His antibody. The concatenated forms of the Esx proteins and many of the similarly linked PPE-PE fusions purified from the soluble fraction as single band products indicating little if any breakdown of the fusion protein at the linker junction (Figure 3B). For example, purified EsxRST and EsxU (Figure 3, panel B, lanes 7 and 8) match those blotted (Figure 2, panel A, lanes 6 and 8) and PPE-PE fusions PPE41_PE25, PPE14_PE7, PPE15_PE8 and PPE20_PE15 (Figure 3, panel B, lanes 10, 14, 16 and 17) matched the proteins identified by blot in crude lysates (Figure 2, panel B, lanes 4, 7, 1 and 2). Sequence concatenation as a viable strategy to reduce clone numbers and to produce single polypeptide versions of multimeric bTB protein complexes, therefore appears feasible, as has been shown for other proteins (reviewed in (Matsushima et al., 2008; van Rosmalen et al., 2017)). Overall, the number of recombinant proteins purified directly without any form of optimisation was ∼50% of the number of clones tested (Table 1), low expression levels or insolubility accounting for the majority of the failures.

### Problematic Clones

**FIGURE 4:**
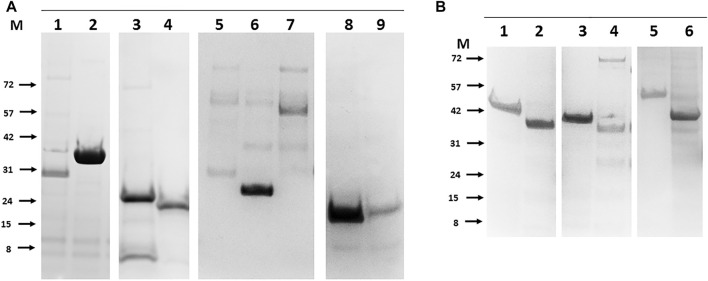
Rescue of soluble purified recombinant bTB proteins that were originally poorly expressed by alternate positioning of the His tag. Panel **(A)**. Examples of N-terminal tagged proteins purified from *E.coli*. 1- Mb3338 (31 kDa), 2-Mb2656 (31 kDa), 3-Mb2898 (22 kDa), 4-Mb2900 (19 kDa), 5-Mb0130 (34 kDa), 6-Mb0854 (31 kDa), 7- Mb1656 (53 kDa), 8-Mb3652 (18 kDa), 9-Mb3871 (20 kDa). Panel **(B)**. Examples of N-terminal tagged proteins purified from insect cells. 1- Mb0977_Mb3046c (43 kDa), 2-Mb0959 (39 kDa), 3-Mb1319 (44 kDa), 4-Mb3070 (37 kDa), 5-Mb2244 (53 kDa), 6-Mb1228 (39 kDa). Molecular weight markers (M) are labelled to the left of each panel and are in kilodaltons.

To improve the overall recombinant bTB protein recovery rate a limited number of ORF sequences were re-cloned as N-terminal His tagged variants and purification repeated. Cell wall proteins (Mb3338 and Mb3070), conserved hypothetical (Mb0854), ribosomal protein (Mb1656), three metabolic proteins (Mb0130, Mb3652 and Mb3871), and six proteins of unknown function (Mb2898, Mb2900, Mb2656, Mb1319, Mb1228 and Mb2244) were all rescued to purified soluble proteins by this approach (Figures 4A,B). Others, such as outer membrane proteins (Mb3644, Mb1445 and Mb2653) remained unworkable. These proteins may require folding partners to be co-expressed in order to be solubilised, as was the case for the original expression trials of PPE-PE family members (Strong et al., 2006).

**FIGURE 5:**
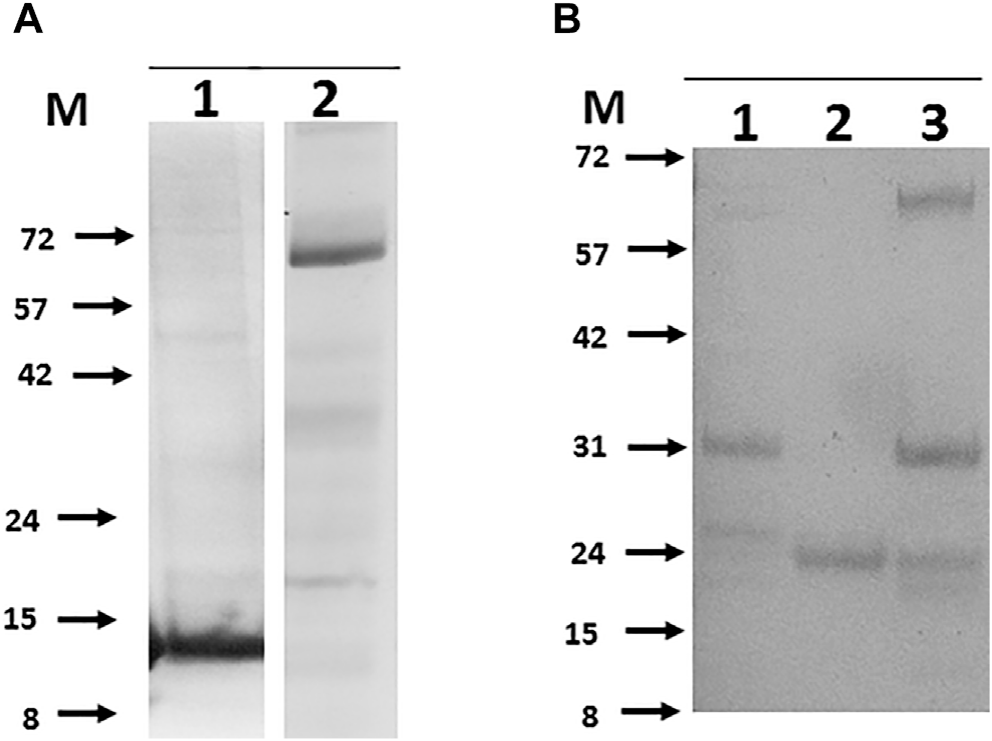
Rescued expression of problematic bTB proteins in *E.coli* by non-His fusion tags. Panel **(A)**. The secretion accessory proteins EspC and EspA. 1- C-terminally His tagged EspC, 2- N-terminally GFP tagged EspA. Panel **(B)**. 1-GB_Mb1762 (29 kDa), 2-GB_Mb1916 (25 kDa), 3-GB_Mb2410 (22 kDa) all expressed and purified as N-terminal fusions with the B1 domain of streptococcal protein G. Molecular weight markers (M) are labelled to left of each panel and are in kilodaltons.

For some problematic proteins the case for their inclusion on grounds of likely candidacy as a vaccine component was such that several alternate forms of expression were assessed. EspA (Mb3646c) has been suggested to be part, with EspC, of the type VII secretion apparatus (discussed in (Ates and Brosch, 2017)) and an obvious candidate for inclusion (Chiliza et al., 2017). Recombinant EspC has been described (Lou et al., 2017; Salemi et al., 2020) and used diagnostically (Yan et al., 2018; Srinivasan et al., 2019; Villar-Hernández et al., 2020), but EspA has proven more difficult (Garces et al., 2010). EspA expression as either a C- or N-terminally His tagged protein was not successful and mutation of the single cysteine reported to drive dimer formation (Garces et al., 2010) had no effect. Fusion of EspA with EspC at either the N- or C- termini via a Gly-Ser linker did not improve expression but, of a number of enhancement tags investigated (GFP, SUMO and GB1), fusion of GFP to the N-terminus rescued detectable expression at the predicted molecular mass in both *E. coli* and insect cells and enabled modest levels to be purified (Figure 5A). Similarly, adding GB1 as an N- terminal fusion tag rescued expression of outer membrane protein Mb1762 and two unknown proteins, Mb1916 and Mb2410, allowing purification to reasonable levels, albeit with contaminating host derived proteins (Figure 5B). To assess if problematic expression was the result of any common feature in the proteins concerned, we inspected the AlphaFold predicted structures (Jumper et al., 2021) of the full length problematic proteins (Jumper et al., 2021) (Table 2). Of the 20 candidates analysed, only 4, Mb2244, Mb2656, Mb3652 and Mb3871 had available full length structures but in each case the proteins concerned were multimeric, some to a very high degree (Mb3871 is a 24mer), which may have limited expression here. Five proteins, Mb1656, Mb1916c, Mb2410, Mb2898 and Mb2900 had partial structures obtained with protein fragments suggesting that full length protein expression was not possible, and a further 5, Mb0130, Mb1228, Mb1319, Mb3070 and Mb3338, had fragment structures predicted on the basis of homology with other proteins whose structures have been solved. Of these 10 proteins, AlphaFold predictions suggested unstructured termini, predominantly at the N-terminus, as a likely limitation to stable protein expression. Unstructured termini, also with a predominance of unstructured N-termini, were associated with the 6 proteins (Mb0854, Mb1445, Mb1762, Mb2653, Mb3644 and Mb3646c) with no available structure, only 3 of which were recoverable here. Submission of the 20 candidate to the FuzDrop server (Hatos et al., 2022) showed a high score for likely aggregation and liquid-liquid phase separation associated with about half of the proteins for which there was either no structure or only a partial structure available (Table 2, indicated). Extensive protein engineering to reduce these scores might be necessary if these proteins were to be produced at a scale and purity required for a vaccine candidate.

**TABLE 2:** Known and predicted structural features of difficult to express proteins.

*M.bovis*	*M.* *tuberculosis*	Structure yes (Y), No (N), homology based (H)	Swiss-model	Alphafold Feature	LLPS Probability
Mb0130	Rv1003	H (2–230 of 285)	3Kwp.1.B	None	0.19
Mb0854	Rv0831c	N		Unstructured N-term	0.2
Mb1228	Rv1196	H (2–175 of 391)	5xfs.1.B	Unstructured C-term	0.944
Mb1319	Rv1288	H (164–447 of 456)	6sx4.1.A	Burried N-term	0.19
Mb1445	Rv1410c	N		Unstructured C-term	0.136
Mb1656	Rv1630	Y (283–438 of 481)	4NNI	Unstructured N-term; highly exteded structure	0.225
Mb1762	Rv1733c	N		Unstructured N-term	0.5
Mb1916c	Rv1884c	Y (68–153 of 176)	4OW1	Unstructured N-term	0.31
Mb2244	Rv2220	Y	1HTQ	None	0.19
Mb2410	Rv2389c	Y (50–127 of 154)	4ow1.1.A	Unstructured N-term	0.54
Mb2653	Rv2620c	N		None	0.22
Mb2656	Rv2623	Y	3CIS	None	0.19
Mb2898	Rv2873	Y (58–219 of 220)	1nyo.1.A	Unstructured N-term	0.63
Mb2900	Rv2875	Y (31–193 of 193)	1NYO	Unstructured N-term	0.17
Mb3070	Rv3044	H (68–352 of 359)	3tny.1.A	Unstructured N-term	0.76
Mb3338	Rv3310	H (5–284 of 299)	1e3c.1.B	Unstructured N-term	0.2
Mb3644	Rv3614c	N		Unstructured N-term	0.46
Mb3646c	Rv3616c	N		Unstructured C-term	0.57
Mb3652	Rv3628	Y	1.WCF	None	0.18
Mb3871	Rv3841	Y	7O6E	None	0.13

## Discussion

Bovine tuberculosis (bTB) is a significant risk to animal health and welfare with estimates of >50 million cattle infected worldwide (Waters et al., 2012). Vaccination with *M. bovis* BCG, which is safe and inexpensive, shows varying efficacies (Francis, 1948; Berggren, 1981; Rodrigues and Smith, 1990) but can reduce spread in cattle indicating that control via vaccination is feasible (Hope et al., 2005; Lopez-Valencia et al., 2010; Waters et al., 2012). However, as up to 80% of BCG-vaccinated cattle may react positively to the tuberculin skin test at 6 months post-vaccination (Whelan et al., 2011), alternate vaccines including new attenuated mycobacterial vaccines (Khare et al., 2007; Waters et al., 2007; Blanco et al., 2013), DNA vaccines (Maue et al., 2004; Cai et al., 2005; Skinner et al., 2005; Maue et al., 2007) and virus-vectored vaccines (Vordermeier et al., 2009; Dean et al., 2014) have been investigated. The reverse vaccinology concept has provided a new serogroup B *Neisseria meningitidis* vaccine, Bexsero (Masignani et al., 2019) and has been used to select vaccine candidates for *Staphylococcus aureus* (Oprea and Antohe, 2013), *Campylobacter jejuni* (Muruato et al., 2017) and *Streptococcus pneumonia* (Argondizzo et al., 2015) in addition to *Mycobacterium tuberculosis* (Monterrubio-López et al., 2015). To test the feasibility for this approach we cloned and expressed >120 ORFs encoded by the reference *M. bovis* AF2122/97 genome and purified more than half of them using a combination of prokaryotic and eukaryotic expression. The list includes Esx protein family members, the PE and PPE families, a number of lipoproteins and others suggested to be potential vaccine candidates, including all of the targets predicted by the VaxiJen package (Monterrubio-López et al., 2015). A useful approach to reduce the number of clones required was to concatenate shorter ORFs or family partners together via flexible linkers. The successful expression and purification of many PPE-PE complexes (PPE4-PE5, PPE68-PE35, PPE69-PE36, PPE65-PE32, PPE41-PE25, PPE17-PE11, PPE15-PE8, PPE14-PE7, PPE31-PE20, PPE20-PE15 and PPE60-PE31) linked in this way was notable and was consistent with the PPE and PE partners folding to form a stable heterodimer (Strong et al., 2006). Many current TB vaccine candidates are fusions of several preselected ORFs (reviewed in (Dockrell, 2016)) and our data suggest that PPE-PE complexes, which have been noted as potential vaccine candidates (Stylianou et al., 2018), may be suitable additions. Currently, those candidate TB vaccines that contain PPE sequences, M72 and ID93, encode only fragments (Brennan, 2017). We did not investigate post-translational modification of any expressed protein as some of these are reportedly *Mycobacteria* specific (van Els et al., 2014) and may not occur in either of the expression hosts used although we noted no particular association with expression outcome. For example the heparin binding hemagglutinin (HBHA), which is methylated and toxic at high levels in *Mycobacteria* (Delogu et al., 2004), was well expression and purified from both *E. coli* and insect cells. We noted some trends in the outcome of expression, e.g. recovery of soluble *rpf*-like proteins (Kana et al., 2008; Mukamolova et al., 2010) was successful from the baculovirus system but not from *E. coli* where they were insoluble, and the combination of these two systems was beneficial overall, providing a success rate for the recovery of purified soluble protein of 67%, slightly higher than the 61% success rate reported during the development of Bexsero (Serruto et al., 2012; Masignani et al., 2019) and considerably higher than the ∼33% success rate previously reported for *Mycobacterial* protein expression using solely *E. coli* (Bashiri and Baker, 2015). An AlphaFold analysis of the proteins where re-cloning with N-terminal tags benefited expression or purification revealed that unstructured termini were a common feature. A tendency to aggregation was also noted by LLPS predication software (Hatos et al., 2022) with the regions identified located in the unstructured regions. Plausibly, the improvements in expression and recoverability for some the targets that were achieved by appending an N-terminal tag stabilised an otherwise disordered structure, reducing aggregation or degradation. A GFP fusion to the sequence encoding full length EspA, where the C-terminal 100 aa of the 319 aa EspA protein sequence has no predictable structure, uniquely allowed expression and purification of this vaccine candidate. A revised HTP scheme (Figure 1) might include an AlphaFold and FuzDrop screen for disorder prior to the selection of the endpoints for translation. That some proteins scored highly for LLPS is interesting given that such regions are associated with stress and lipid homeostasis (Vendruscolo, 2022), both of which are hallmarks of TB infection (Chow and Cox, 2011). Notwithstanding these considerations, our data suggest that the production of a large library of full length bTB proteins for candidate vaccine use is feasible. Recently the protection afforded by intravenous vaccination with BCG was shown to be correlate with the IgM component of the antibody response, although no antigen specificity was reported (Divangahi et al., 2021; Irvine et al., 2021). In addition, a monoclonal antibody to a single protein, PstS1 (Mb0959 in Table 1, purified in Figure 4B), was inhibitory in a whole blood Mtb growth inhibition assay (Watson et al., 2021). Antibodies from animals immunised with proteins from the range we describe here might be used to identify other targets relevant for vaccine development against Bovine TB or the related *Mycobacterium tuberculosis* and *Mycobacterium leprae*.

## Data Availability

The original contributions presented in the study are included in the article/Supplementary Material, further inquiries can be directed to the corresponding author.
